# Transcriptome Analysis of Male and Female *Sebastiscus marmoratus*

**DOI:** 10.1371/journal.pone.0050676

**Published:** 2012-11-27

**Authors:** Lingbin Sun, Chonggang Wang, Lixing Huang, Meifang Wu, Zhenghong Zuo

**Affiliations:** 1 State Key Laboratory of Cellular Stress Biology, School of Life Sciences, Xiamen University, Xiamen, China; 2 State Key Laboratory of Marine Environmental Science, Xiamen University, Xiamen, China; Ecole Normale Supérieure de Lyon, France

## Abstract

**Background:**

The rockfish *Sebastiscus marmoratus*, which is widely distributed in the East Sea and the South Sea of China, is a sensitive model for the toxic effects and mechanisms of marine contaminants. To gain a global view of the molecular mechanism(s) whereby gene expression may influence sexual dimorphism in *S. marmoratus*, and to develop a database for further toxicological studies, we performed a large-scale transcriptome study.

**Methodology/Principal Findings:**

The Illumina DNA sequencing platform was employed to obtain 27,559,578 and 25,821,126 reads from two cDNA libraries generated from adult male and female *S. marmoratus*, respectively. Transcriptome *de novo* assembly was carried out with the short reads assembling program–SOAPdenovo. The reads assembled into 78,675 unigenes, of which 38,677 showed homology to existing protein sequences. Clusters of orthologous groups, gene orthology, and the Kyoto Encyclopedia of Genes and Genomes annotations were performed to better understand the functions of these unigenes. There were 1,209 potential sex differentially expressed unigenes, with 1,049 predicted to be differentially expressed in females and 160 in males. Fifteen randomly chosen unigenes were confirmed using real-time PCR as female or male predominantly expressed genes.

**Conclusions/Significance:**

This is the first report of an annotated transcriptome of *S. marmoratus* and identification of sex differentially expressed genes. These data will be of interest to researchers using this model. This work also provides an archive for future studies in molecular mechanisms of sexual dimorphism and evolution and can be used in comparative studies of other fish.

## Introduction

Over the last 100 years manufacturing industries have produced and introduced into the environment a plethora of both organic and inorganic contaminants. These contaminants are discharged into rivers, lakes and the ocean in the aquatic environment and are spread between water columns (interstitial waters which interface with the sediment) and a solid phase (sediment and particulates in suspension) [1]. High lipophilicity combined with chemical persistence and biomagnification in the food chain are characteristic features of persistent organic pollutants (POPs) [2]. With the biomagnification of POPs in the aquatic food chain, the high concentrations reached at the top of the food-web may exceed toxicity thresholds, triggering biochemical disturbances and physiological changes in the contaminated individuals [3]. Ultimately, high-level predators may be subjected to harmful concentrations of contaminants. Thus, effective detection of contaminants requires complementary information regarding model organisms.

In the past, marine mussels were selected for the study of coastal pollution impacts on marine life [4]. However, marine mussels belong to the lower reaches of the food chain. More recently, the marbled rockfish, *Sebastiscus marmoratus,* an ovoviviparous fish inhabiting littoral rocky bottoms has been selected as a potential model organism for testing pollutants toxicity [5]–[6]. Generally, the rockfish attains a length of 15–20 cm, while some individuals can grow up to 30–40 cm. *S. marmoratus* inhabits littoral rocky bottoms and migrates within oceans typically between different spawning and feeding areas. *S. marmoratus* was used as a selected organism since the rockfish offers several advantages for these studies. Firstly, *S. marmoratus* is a demersal fish that is found near the shore, on rocky bottoms [7], and is widely distributed in the East Sea and the South Sea of China, and from southern Japan to eastern Korea [8]. Secondly, *S. marmoratus* is a viviparous fish providing thousands of 4 mm eggs from winter to spring. These eggs are fertilized internally, retained, and undergo development in the maternal reproductive system [9]. Thirdly, as an ambush predator feeding on shrimps, crabs and fishes, the rockfish represents a higher trophic level [10]. Fourthly, the rockfish is an important commercial food, and so it is important to prevent exposure to chemicals which might affect the reproduction and general health of the fish, as well as its suitability as a food for humans. Together with its well characterized biomagnification process, the rockfish is sensitive to chemical contaminants and thus the effects of many chemicals can be exhibited quickly. These attributes make *S. marmoratus* an appropriate model for toxicological studies. Progress in molecular biology over the past decade has revealed that *S. marmoratus* is a sensitive model for both the toxic effects and the mechanisms of marine contaminants [11]–[14]. Our previous study proved that the male and female fish have different toxicity in response to chemicals (data not shown). However, there is a need to further elucidate the molecular mechanism of these differences which are induced by chemical exposure, including knowledge of gender differences in the levels of altered gene expression.

Traditionally, the field of toxicology focuses on assessing potential adverse health effects resulting from chemical exposure by using gross endpoints such as morphological changes and histopathological observations [15]. However, the evolution of new, innovative technologies coupled with data analysis is revolutionizing toxicological science. Recent research has shown that high-throughput RNA-sequencing (RNA-Seq) technology is a powerful, cost-efficient tool for transcriptome analysis and provides a more sensitive tool than microarray methods [16]–[17]. Besides, the use of RNA-Seq technology provides general representation of almost all the transcripts expressed in specific organs at particular conditions and times [18]. The genome and transcriptome data for many vertebrate species, particularly marine fishes, have not yet been disclosed [19]. To our knowledge, a limited number of *S. marmoratus* genes have been cloned and characterized, based on bioinformatic analysis, including those involved in lipid metabolism and in testicular and skeletal development. Use of RNA-Seq technology may help us understand the dissimilar mechanisms at the RNA level. Obviously, large scale transcriptome analyses have great potential to identify the gene expression changes through a comparison of male and female samples. In the present study, we investigated the transcriptome of male and female *S. marmoratus* and our objectives were: (1) to develop a transcriptome database of male and female *S. marmoratus* for further toxicological studies; and (2) to gain a global view of the molecular mechanism(s) whereby gene expression may influence sexual dimorphism in *S. marmoratus*.

## Results

### Sequencing and assembly of *S. marmoratus* cDNAs

In order to capture as many transcripts of *S. marmoratus* as possible, two cDNA libraries were constructed from RNA isolated from female and male samples. Sequencing runs were performed on the male and female cDNA libraries generating 27,559,578 and 25,821,126 reads, respectively (All the sequence reads are published in GenBank with accession number SRP010778). Approximately 93.62% and 93.56% of reads passed the quality required. Transcriptome *de novo* assembly was carried out with the short reads assembling program SOAPdenovo [20]. Unigenes with a length of 300 bp made up the majority of the assembled unigenes. The size distribution of the assembled unigenes is presented in Figure S1. About 49% of assembled unigenes could be annotated using the public databases (Table 1). Others might correspond to untranslated regions, non-coding RNAs, or short sequences not containing known protein domains. Such a high percentage of novel genes might justify a deep sequencing coverage since it seemed likely that “novel” ESTs would be found in more rarely expressed genes.

**Table 1:** Overview of transcriptome analysis.

Total number of reads	53,380,704
number of reads from male	27,559,578
number of reads from female	25,821,126
number of unigenes from assembly	78,675
number of unigenes from male	86,130
number of unigenes from female	93,537
number of known genes (%)	49%
number of unknown genes (%)	51%
Among the unigenes, Enriched with FDR≤0.001 AND |log2Ratio|≥6.64	
male enriched unigenes	160
female enriched unigenes	1049
number of known enriched genes (%)	51%
number of unknown enriched genes (%)	49%

### Functional classification of predicted proteins

The reads assembled into 78,675 unigenes, the sequences of which contained determinable direction. All of the unigenes were used for homology searching against the nonredundant (nr) NCBI nucleotide database by performing BLASTX with a cut-off E value of the best hit of ≤10^−5^. The top-hit species in the annotated distribution was *Danio rerio* (57%) followed by *Salmo salar* (10%). The distribution tendency of the E values upon the annotated unigenes is displayed in Figure S2. More than 90% of the annotated unigenes had an E value<10^−10^, which gave an indication of the reliability and validity of our annotated results based on the databases available. There were 38,677 unigenes that shared homology to existing protein sequences. Except for those sequences with undeterminable direction, there were 51% unigenes that had no database hits, which possibly represented unigenes with unknown functions.

**Figure 1:**
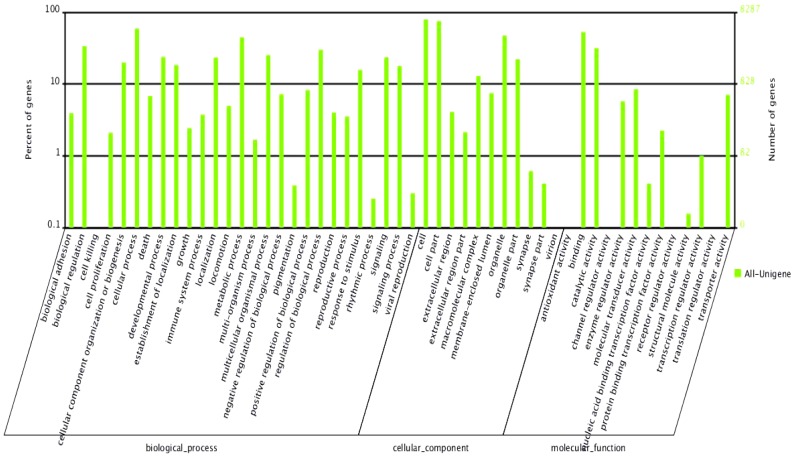
Functional annotation of unigenes based on known proteins in the Uniprot database. Each annotated sequence was assigned at least one GO term. GO terms at the second level were displayed to classify the results based on their involvement in biological processes, molecular functions, and cellular components.

The putative functions of unigenes in two different libraries were analyzed according to gene ontology (GO) and clusters of orthologous groups (COGs) of protein classifications. Analysis of GO categories showed that the functional distribution of the genes of the two libraries was similar. There were 8,287 unigenes annotated with 62,079 GO terms. A total of 31,986 unique sequences mapped to biological processes, 20,949 to cellular components, and 9,144 to molecular functions in the assembly of male and female libraries. In the libraries, most of the corresponding biological process genes were involved in cellular processes, biological regulation and metabolic processes. Most of the cellular component genes encoded proteins associated with cells and cell parts; and most of the molecular function genes were associated with binding and catalytic activity (Figure 1).

**Figure 2:**
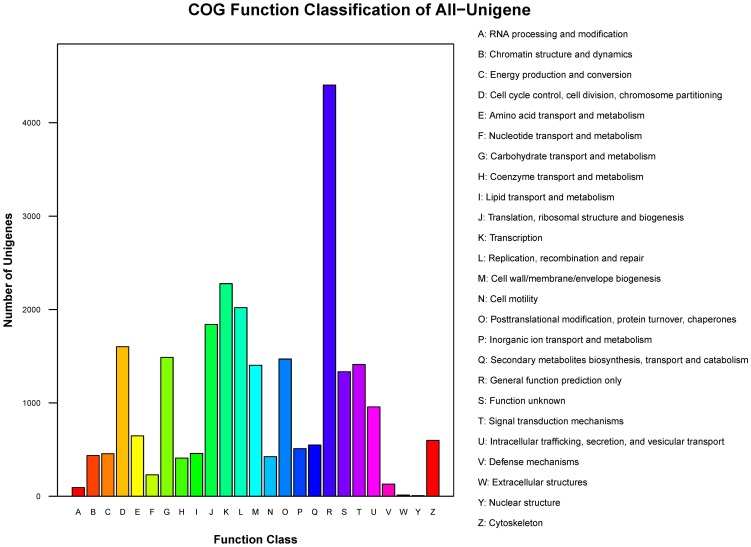
Histogram presentation of clusters of orthologous groups (COGs) classification of all-unigenes.

The COGs database represents an attempt at phylogenetic classification of the proteins encoded in complete genomes [21]. Among the 25 COGs categories, the cluster in the assembly of male and female libraries for “General function prediction only” represented the largest group (4403 unigenes), followed by the “Transcription” and “Replication, recombination and repair” clusters (Figure 2).

To identify the biological pathways that are active in sexual dimorphism, we mapped the unigenes to reference canonical pathways in the Kyoto Encyclopedia of Genes and Genomes (KEGG). Using KEGG, 26,093 unigenes were assigned to 219 KEGG pathways. Those pathways with the greatest representation by unigenes were pathways for metabolic functions (2,668 unigenes), regulation of the actin cytoskeleton (1,234 unigenes), pathways in cancer (1,198 unigenes) and focal adhesion (1,037 unigenes). These annotations provided a substantial resource for investigating specific processes, functions, and pathways during sexual dimorphism.

### Sex-enriched gene expression and confirmation using real-time PCR

Poisson-based enrichment testing identified 160 male-enriched and 1049 female-enriched unigenes (Table 1) with various degrees of difference. These unigenes showed significant changes in transcriptional expression (i.e. exhibited at least 100-fold enrichment of sequences in one sex over the other). Among these enriched unigenes, there were 51% unigenes that had annotation. The up-regulated and down-regulated unigenes between male and female libraries are listed in Table S2. To validate statistically whether the genes were up-regulated or down-regulated, we detected the relative expression of sex-enriched genes using real-time PCR. Among these unigenes, 11 female-predominant transcripts and four male predominant unigenes were confirmed as sex differentially expressed genes (Table S3).

## Discussion

Transcriptome sequencing using next-generation technologies provides resources for gene expression profiling studies as well as simultaneous identification of mutations, sequence aberrations, alternative splice variants, and RNA editing events [22]. The present study focused on applications of next-generation sequencers to transcriptome analysis of *S. marmoratus* and further toxicology research. One of the most important aspects in transcriptome analysis is to associate individual sequences and related expression information with biological functions. These annotations offer a valuable resource for further research related to sexual dimorphism and toxicology.

In order to detect differentially expressed genes, we established an adult male and female model as the RNA source. The RPKM (reads per kb per million reads) value was employed to eliminate the influence of different gene length and sequencing level on the calculation of gene expression. Thus, the sex-enriched genes could be identified by comparing read counts from male and female. It was obvious that sex differentially expressed genes were uniquely or predominantly expressed in female. The developing oocyte is surrounded by an acellular envelope that is composed of zona pellucida (ZP) proteins [23]. Among these sex-enriched unigenes, several “oocyte” related genes, such as ZP, were predominantly expressed in the female. Enzymes present in the oocyte catalyze a series of metabolic processes vital for the production of viable offspring. They include enzymes such as the cathepsins that degrade vitellogenin into yolk proteins for storage in the oocyte and, post-fertilization, which mediate the degradation of the stored yolk proteins into free amino acids for use by the developing embryo [24]–[25]. Our results demonstrated that the cathepsins were mainly expressed in females. From transcriptome analysis, several ‘hormone” related genes were found in those unigenes which showed significant differences in male and female. Anti-Müllerian hormone, also known as Müllerian inhibiting substance, is a member of the transforming growth factor superfamily of growth and differentiation factors [26]. Follicle stimulating hormone (FSH) is considered essential for folliculogenesis in the female and spermatogenesis in the male. FSH is predominately expressed in the female rather than the male, which demonstrates that FSH is more important for female than for male fertility. Luteinizing hormone is also critical for female fertility [27].

Male and female fish have wide variations in their response to chemicals [28]. Although the molecular mechanism of these differences which are induced by chemical exposure is still ambiguous, we thought that the sex-biased genes can lead the different level of tolerance to toxins between sexes. For example, a Na^+^/bile acid transporter, known as a transporter of both Na^+^ and bile acid, is predominantly expressed in male. Defects in bile acid transporters cause progressive familial intrahepatic cholestasis [29]. It may be a possible reason to cause the different level of tolerance to toxins between sexes for detoxification.

We chose 15 genes randomly for validation using real-time PCR and the results were in accordance with transcriptome analysis. Those selected unigenes such as vitellogenin, Mullerian inhibiting substance, zygote arrest protein 1 and Cathepsin Z precursor showed significant differences between males and females. Although deep sequencing succeeded, as confirmed by real-time PCR, the differences between males and females were different between the deep sequencing and the real-time PCR validation. The reason for this is probably because the values of RNA were different: RNA for deep sequencing was isolated from pooled fish organs while the RNA for RT-PCR was isolated from dissected organs. We also found that the expression levels of sex differentially expressed genes were different in different organs. Combining real-time PCR test and annotations, many differentially expressed unigenes were specifically expressed in the gonad, while some were focused on liver. Further studies of sex differentially expressed genes in the list are required to test whether they provide critical clues to resolve the problem of sexual dimorphism and sex difference. There is also potential for the discovery of new genes in this organism and possibly of new gene networks and metabolic pathways using the assembled data. This is the first large dataset available for further analysis of *S. marmoratus*.

### Conclusion

As the first report of an annotated overview of *S. marmoratus* and identification of sex differentially expressed genes, this research described the application of the next generation sequencing technology to facilitate discovery of sex differentially expressed genes. This gene information provides further insights into sexual dimorphism and sex difference using the *S. marmoratus* transcriptome.

## Materials and Methods

### Ethics statement

Animal experiments were carried out in strict accordance with the recommendations in the Guide for the Care and Use of Laboratory Animals. The protocol was approved by Xiamen University Institutional Animal Care and Use Committee (XMUEA-0080). All efforts were made to minimize suffering.

### Experimental species

Male and female *S. marmoratus* weighing 20–30 g were captured from a pristine coast in Xiamen City, Fujian Province, China. In order to increase the representation of the transcriptome, nonpregnant females (without embryos in these fish) were chosen for total RNA isolation from whole fish for sequencing. Before the sample collection, male and female fish were acclimated in two tanks (eight fish in each tank) containing 30 L of aerated sand-filtered seawater, under flow-through conditions with natural photoperiod for 7 days. The water temperature was maintained at 14±2°C and salinity 22–24‰. There was no fish died during this period. At the end of period, six fish from each group were randomly selected and anesthetized with 0.06% ethyl 3-aminobenzoate methanesulfonate salt (MS-222). The tissue was dissected out, weighed and placed immediately in liquid nitrogen, and stored at −80°C until use. Six male and six female fish were dissected to obtain samples of brain, liver, heart, pancreas, spleen and gland, and these samples of the female and male were used for RNA isolation to be employed in sequencing and real-time PCR validation studies.

### RNA isolation

The tissue sample pool was obtained according to the following procedure; each tissue from the six male or female fish was put in a homogenizer in an ice bath by adding 1.5 mL Trizol and 100 mg tissue, rapid grinding, and equal volume of the homogenate loaded on a tube. Total RNA was extracted from fish tissues using a Trizol Kit (TaKaRa, Dalian, China) according to the manufacturer's procedure. The quantity of RNA isolated was determined by measuring the optical density in a SmartSpec™ Plus spectrophotometer (BIO-RAD, USA) at 260 nm and its purity was established by calculating the absorbance ratio 260/280 nm (1.8–2.0). The quality of RNA was examined using 1.2% agarose gel electrophoresis.

### cDNA library construction and Illumina sequencing

Based on the method of Shen et al. [30], 12 µg total RNA (a mixture of RNA from the brain, liver, heart, pancreas, spleen and gland in equal ratios) was used to construct a cDNA library. Poly (A) mRNA was purified from total RNA using oligo (dT) magnetic beads. It was then fragmented into small pieces by the addition of fragmentation buffer. These short fragments served as templates to synthesize first-strand cDNA using random hexamer-primers. Second-strand cDNA was synthesized using buffer, dNTPs, RNaseH and DNA polymerase I. Short fragments were purified using a QiaQuick PCR extraction kit. These fragments were washed with EB buffer for end reparation poly (A) addition and then ligated to sequencing adapters. Suitable fragments, as judged by agarose gel electrophoresis, were selected for use as templates for PCR amplification. The cDNA library was sequenced on an Illumina HiSeqTM 2000 using paired-end technology in a single run. The raw tag sequence data were analyzed for gene annotation, genome annotation, and functional annotation. The quality of all steps was controlled in accordance with the recommendations of Illumina.

### Analysis of function

All the unigenes from the contigs assemblied from the RNA-Seq short reads were submitted for homology and annotation searches, and GO annotation using an online version of the BLAST2GO program (www.Blast2GO.de; Oct. 2009) [31]. In these searches the BLASTX cut off value was set to 10^−5^. The annotation step of the program retrieves keywords in the BLASTX descriptions and converts them into GO related terms associated with homologies identified with NCBI's QBLAST and returns a list of GO annotations represented as hierarchical categories of increasing specificity. Placement into metabolic pathways was accomplished with tools supplied by the KEGG (Oct., 2009), located at the KEGG Automatic Annotation Server (KAAS), http://www.genome.jp/kegg/kaas/. The output includes KO (KEGG Orthology) assignments and automatically generated KEGG pathways that are populated with the KO assignments. Annotation results were then used to retrieve keywords to identify genes related to sex differentiation.

### Identification of male and female differentially expressed genes

A Poisson-based enrichment test, considering both the total sampling sizes and random variations [32], was utilized to calculate the likelihood of gender specific enrichment for each unigene. The test identifies differential expression from cDNA profiles by calculating the probability of counts from one sex when the counts from the other sex are known, with the assumption that the counts originated from the same distribution. In order to calculate the unigene expression levels, the RPKM value was measured [33]. The RPKM measure of read density reflects the molar concentration of a transcript in the starting sample by normalizing for RNA length and for the total read number in the measurement. The RPKM value is able to eliminate the influence of different gene length and sequencing level on the calculation of gene expression, and therefore, the calculated gene expression can be directly used for comparing the difference of gene expression between samples. We then used the FDR (false discovery rate) method [34] to determine the threshold of the P-value in multiple tests. In this study, we used ‘FDR≤0.001 and the absolute value of log2Ratio ≥100’ as the threshold to judge the significance of unigene expression.

### Quantitative real-time PCR (qRT-PCR) analyses

The primers (listed in Table S1) were designed using the Primer Premier 5.0 program (PREMIER Biosoft International, Silicon Valley, CA). Identification of the sequences was carried out using basic local alignment search tool (nucleotide BLAST) analyses against National Center for Biotechnology Information (NCBI) data (http://www.ncbi.nlm.nih.gov/BLAST/).

To perform RT-PCR, DNA free *S. marmoratus* RNA from brain, liver and gland of four female and four male fish was reverse-transcribed using 1 µg total RNA of each group and a PrimeSript™ 1st Strand cDNA Synthesis Kit (TaKaRa, Dalian, China) according to the manufacturer's procedure. The cDNA samples obtained were diluted 1∶10 with sterile water before their use as templates in real-time quantitative PCR.

Levels of mRNA were determined using qRT-PCR and SYBR Green chemistry on a Stratagene Mx3000P (Agilent Technologies, USA). The mRNA expression of these genes in the brain, liver and gland was detected based on the method used in our previous study [35]. The Relative Expression Software Tool (REST 2008©-version 2) was used to calculate the relative expression of target mRNA. Results are reported as mean ± standard error (S.E.). Significant differences between means were analyzed with the Pair Wise Fixed Reallocation Randomization Test© [36]. In all cases, a value of *P*<0.05 was used to indicate significant differences.

## Supporting Information

Figure S1 **Size distribution of assembled unigenes.** Transcriptome *de novo* assembly was carried out with the short reads assembling program – SOAPdenovo. Unigenes with a length of 300 bp occupied the majority of assembled unigenes.
(TIF)

Figure S2 Distribution of E-values (a) and top-hit species (b) from the top hit in the non-redundant protein database.
(TIF)

Table S1 **Primer for real-time PCR.**
(DOC)

Table S2 **List of the up-regulated and down-regulated unigenes between male and female libraries.**
(XLS)

Table S3 **Real-time PCR confirmation of sex differential expressed genes analyzed with the Pair Wise Fixed Reallocation Randomization Test© and based on annotation (successfully validated).**
(DOC)
