# Supplementary figures and images for: Transcriptome Analysis of Male and Female Sebastiscus marmoratus
Source: PLoS One. 2012 Nov 27;7(11):e50676. doi: 10.1371/journal.pone.0050676 (PMC3507777; doi:10.1371/journal.pone.0050676)

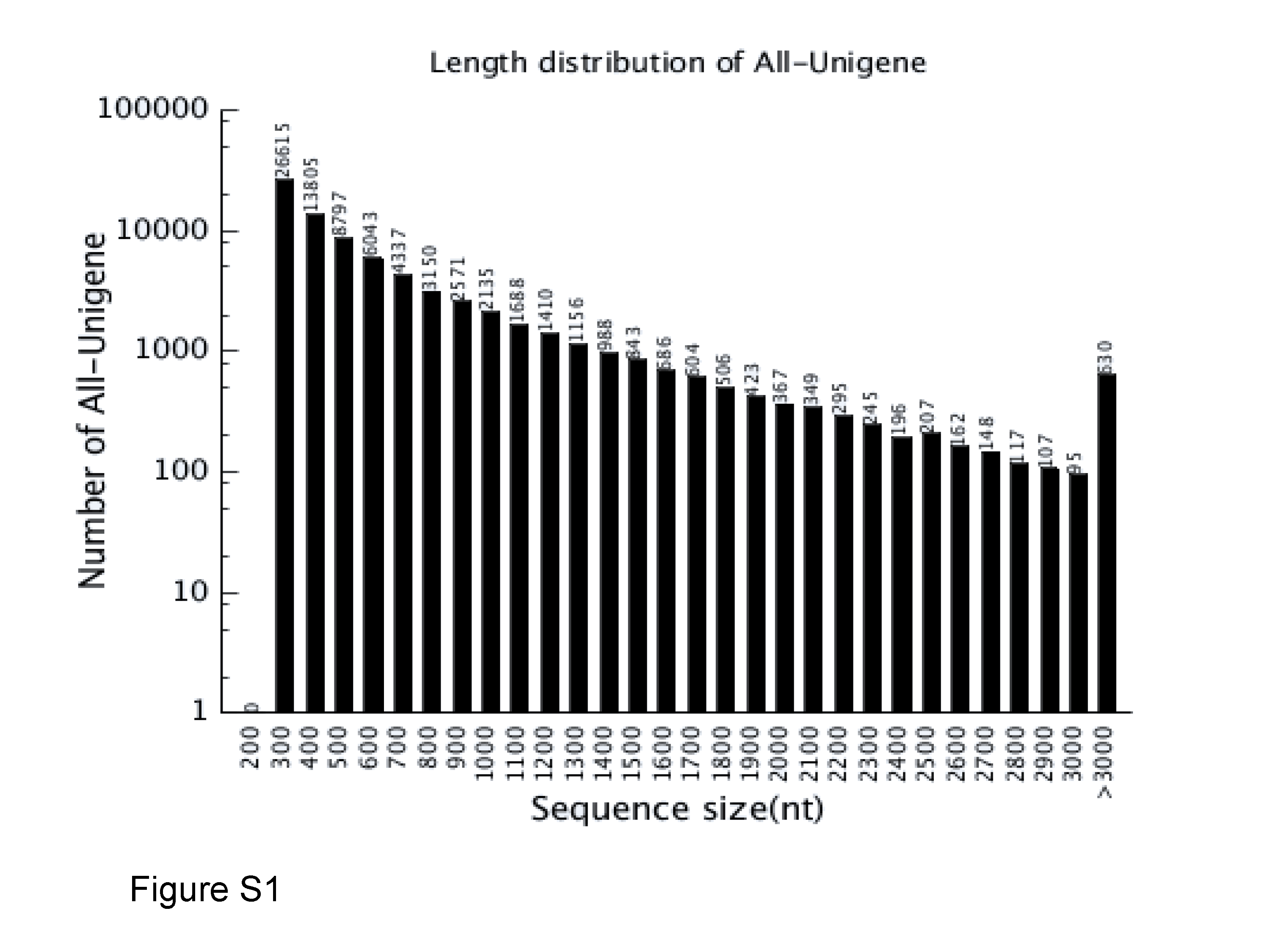

Supplement: Figure S1 — Size distribution of assembled unigenes. Transcriptome de novo assembly was carried out with the short reads assembling program – SOAPdenovo. Unigenes with a length of 300 bp occupied the majority of assembled unigenes. (TIF) [file pone.0050676.s001.tif]

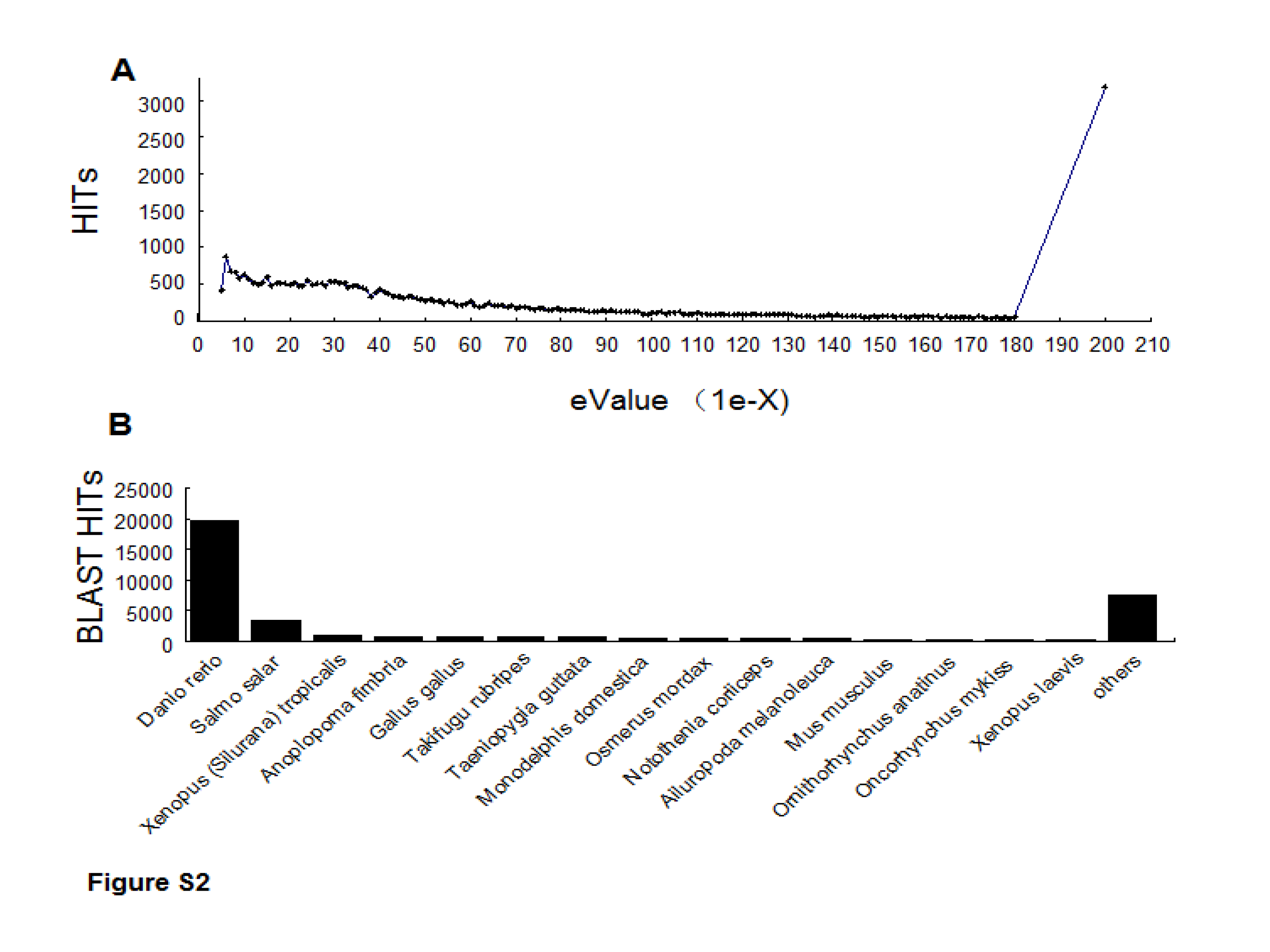

Supplement: Figure S2 — Distribution of E-values (a) and top-hit species (b) from the top hit in the non-redundant protein database. (TIF) [file pone.0050676.s002.tif]
