# Supplementary material for: Transcriptome Analysis of Male and Female Sebastiscus marmoratus
Source: PLoS One. 2012 Nov 27;7(11):e50676. doi: 10.1371/journal.pone.0050676 (PMC3507777; doi:10.1371/journal.pone.0050676)
Supplement: Table S1 — Primer for real-time PCR. (DOC) [file pone.0050676.s003.doc]

**Table S1 Primer for real-time PCR**

| ID of Primer | Sequence of primer |
| --- | --- |
| unigene60278F: | 5'- AAAGGGATGTGAAGGACTGGA -3' |
| unigene60278R: | 5'- AGGACAGGCGGAAGGAGTG -3' |
| unigene77219F: | 5'- TAAACAGCTTCACCTCCAGAC -3' |
| unigene77219R: | 5'- TCTTGTACCTCCACTCTTTAGC -3' |
| unigene77364F: | 5'- GCTGACTCTGCTGGTTGATA -3' |
| unigene77364R: | 5'- CAGGATGGATTCATGGCTT -3' |
| unigene83085F: | 5'- GACGACCGCTTCGCTTATTT -3' |
| unigene83085R: | 5'- GCCCTGAACCGATCTGTGC -3' |
| unigene83265F: | 5'- CTGAGGATGTGGAGGAACTAC -3' |
| unigene83265R: | 5'- TGTATGGAGGCAGGAAGAAC -3' |
| unigene78571F: | 5'- GGATAATGGAGAACCGCAAAG -3' |
| unigene78571R: | 5'- GGGTCGCAGTGAAAGGTGTAT -3' |
| unigene83866F: | 5'- ACGAGGACACGAGATACAAAG -3' |
| unigene83866F: | 5'- AAGGTGATGTTCATTAGAGCC -3' |
| unigene57363F: | 5'- CGCACAGACAGCGAAGTAT -3' |
| unigene57363R: | 5'- CTCTGTTGATGGGAAGGTGT -3' |
| unigene80788F: | 5'- TACGTCGAGACACCGGCAAT -3' |
| unigene80788R: | 5'- CACAATCTTGAGCCAGCCTTTC -3' |
| unigene9844F: | 5'- CAGAAGGGCCTCAAAGATT -3' |
| unigene9844R: | 5'- GCCGTGTAGTCCATGTAGTG -3' |
| Unigene60527F: | 5'- TCAACATCAACAACTGCCACG -3' |
| Unigene60527R: | 5'- CGTTCAAGTCCACCACCTCC -3' |
| Unigene43553F: | 5'- GGTCCTCTGTTCATGTTCCC -3' |
| Unigene43553R: | 5'- CCGGCCTACTACCTTTCTT -3' |
| Unigene83819F: | 5'- CCTCAAGTCCAACCTCCAAAT -3' |
| Unigene83819R: | 5'- TGTTCCCGACACTGCACCT -3' |
| Unigene52879F: | 5'- CCACATCTGCCAAGACCAT -3' |
| Unigene52879R: | 5'- CAGCTCGTTAGTGCAATCCT -3' |
| Unigene60441F: | 5'- GCCAGCCTTTGGTGATTGAG -3' |
| Unigene60441R: | 5'- GGCGATGGTCAGAGGGATG -3' |
| 18s-F: | 5'- GGGTCCGAAGCGTTTACT -3' |
| 18s-R: | 5'- CACCTCTAGCGGCACAATAC -3' |
