# Supplementary material for: Transcriptome Analysis of Male and Female Sebastiscus marmoratus
Source: PLoS One. 2012 Nov 27;7(11):e50676. doi: 10.1371/journal.pone.0050676 (PMC3507777; doi:10.1371/journal.pone.0050676)
Supplement: Table S3 — Real-time PCR confirmation of sex differential expressed genes analyzed with the Pair Wise Fixed Reallocation Randomization Test© and based on annotation (successfully validated). (DOC) [file pone.0050676.s005.doc]

**Table S2 Real-time PCR confirmation of sex differential expressed genes** **analyzed with the Pair Wise Fixed Reallocation Randomization Test© and based on annotation (successfully validated)**

| Unigene ID | #female reads * | #male reads * | Ovary verse Testis | | Female Liver verse Male Liver | | Female brain verse Male brain | | Nr-annotation | Nr-Evalue |
| --- | --- | --- | --- | --- | --- | --- | --- | --- | --- | --- |
|  |  |  | Fold | P value | Fold | P value | Fold | P value |  |  |
| 60278 | 1546 | 2 | 10.23 ± 6.39 | 0.007 | No difference |  | No difference |  | bone morphogenetic protein 15 | 0 |
| 77219 | 460 | 0 | 9.62 ± 5.90 | 0.02 | 2.22 ±1.71 | 0.042 | No difference |  | zygote arrest protein 1 | 3.00E-40 |
| 77364 | 474 | 0 | 15.54 ± 9.78 | 0.023 | 3.64 ± 5.60 | 0.044 | No difference |  | zona pellucida C related protein | 1.00E-11 |
| 83085 | 2114 | 4 | 10.81 ± 7.00 | 0.019 | No difference |  | No difference |  | ZPA domain containing protein | 1E-103 |
| 83265 | 463 | 0 | 11.45 ± 7.03 | 0.02 | No difference |  | No difference |  | small nuclear RNA activating complex, polypeptide 1a | 3.00E-46 |
| 78571 | 2355 | 5 | 15.42 ± 9.87 | 0.000 | 1.97 ± 1.38 | 0.002 | No difference |  | elongation of very long chain fatty acids protein 7 | 1.00E-130 |
| 83866 | 1213 | 0 | 11.14 ± 6.72 | 0.026 | No difference |  | No different |  | zona pellucida protein X | 0 |
| 57363 | 0 | 618 | -2.69 ± 0.05 | 0.016 | No difference |  | No difference |  | anti-Mullerian hormone | 8.00E-59 |
| 80788 | 1965 | 0 | 15.08 ± 8.75 | 0.001 | No difference |  | No difference |  | Cathepsin Z precursor | 4.00E-40 |
| 9844 | 9647 | 59 | 14.36±9.26 | 0.034 | No difference |  | No difference |  | cyclin B1 | 1E-110 |
| 60527 | 9 | 576 | -2.42 ± 0.04 | 0.006 | No expression |  | No expression |  | Mullerian inihibiting substance | 1.00E-54 |
| 43553 | 0 | 131 | -2.42 ± 0.04 | 0.034 | No difference |  | No difference |  | PREDICTED: sodium/bile acid cotransporter isoform 2 | 2.00E-10 |
| 83819 | 793 | 1 | 12.95 ± 8.06 | 0.011 | No difference |  | No difference |  | ubinuclein-1 [Gallus gallus] | 8.00E-82 |
| 52879 | 46010 | 14 | 4.27 ± 1.26 | 0.001 | 16.52 ±5.67 | 0.001 | No difference |  | vitellogenin | 7.00E-34 |
| 60441 | 0 | 1106 | -4.80 ± 0.01 | 0.004 | 3.079 ± 2.30 | 0.007 | No difference |  | alcohol dehydrogenase Class VI | 6.00E-69 |
